# Domestic Violence and Education: Examining the Impact of Domestic Violence on Young Children, Children, and Young People and the Potential Role of Schools

**DOI:** 10.3389/fpsyg.2018.02094

**Published:** 2018-11-13

**Authors:** Michele Lloyd

**Affiliations:** The School of Education, University of Hertfordshire, Hatfield, United Kingdom

**Keywords:** domestic violence, education, early childhood, children, young people, schools, teachers, multi-agency working

## Abstract

This article examines how domestic violence impacts the lives and education of young children, children, and young people and how they can be supported within the education system. Schools are often the service in closest and longest contact with a child living with domestic violence; teachers can play a vital role in helping families access welfare services. In the wake of high profile cases of child abuse and neglect, concerns have been raised about the effectiveness of multi-agency responses to children living with abuse. In the United Kingdom, the case of 4-year-old Daniel Pelka who died in 2012 following abuse and starvation by his mother, who experienced domestic violence, and her partner, led to a serious case review. It found recording systems in Daniel’s school were not used consistently, and details held by different agencies were not collated to enable the formation of a coherent assessment. The lack of integrated working cited in the report echoes findings from previous serious case reviews. A strong correlation exists between domestic abuse and child abuse, with approximately half of all domestic violence situations involving direct child abuse. Children can also be affected indirectly by violence occurring in their home by seeing or hearing it taking place. This article examines the impact of domestic violence on the mental health of children, and the impact on their education. Violence in children’s lives often causes disruption to their schooling and harms the quality of their educational experiences and outcomes. The abuse children experience can result in emotional trauma, physical and psychological barriers to learning, and disruptive behavior in school, while the underlying causes of these problems remain hidden. Knowing when and how to seek advice from multi-agency professionals is an essential part of effective practice among school staff. Despite their vital role in identifying signs of abuse and signposting referral pathways, research indicates teachers often lack confidence and knowledge for such work. The article examines how the professional learning and professional confidence of teachers can be developed, and how recent policy and practice developments in the United Kingdom have the potential to influence work in this area.

## Introduction

Every school is likely to have children affected by domestic violence. The aim of this article is to examine how domestic violence impacts the lives of young children, children, and young people, and the potential role that schools can play in helping to address their needs. Wellbeing and healthy relationships are the foundations of learning. The immediate and long-term costs of domestic violence can thus be high, affecting children’s education as well as having long-term developmental consequences.

Many types of abuse occur within the domestic sphere. In the United Kingdom the government definition of domestic violence and abuse is: ‘any incident or pattern of incidents of controlling, coercive, threatening behavior, violence or abuse between those aged 16 or over who are, or have been, intimate partners or family members regardless of gender or sexuality. The abuse can encompass, but is not limited to: psychological, physical, sexual, financial, emotional’ (Home Office, 2018). Although this definition applies to those aged 16 or over, children also experience the harmful effects of domestic violence, as will be examined further below. There is growing awareness of the emotional harm of domestic violence, evidenced in the United Kingdom in the offense of controlling or coercive behavior in intimate or familial relationships which has a maximum custodial sentence of 5 years, a fine or both (Home Office, 2015). As well as being affected by physical abuse, children can be affected by non-physical domestic abuse based on coercive control, such as isolation, continual monitoring, financial abuse, and verbal and psychological abuse (Katz, 2016). Domestic violence is part of the landscape of child protection. Government documentation on child protection titled ‘Working Together to Safeguard Children’ (HM Government, 2013) details safeguarding responsibilities of professionals and organizations, and promotes a child-centered approach based on the needs and views of children (Holt, 2014).

While the term domestic violence is used in this article, a number of terms are present in literature such as intimate partner violence and inter-parental violence, and sometimes terms are used interchangeably. Since there is a governmental definition of domestic violence, and also for consistency, I will adopt the term domestic violence predominantly here. The terms intimate partner violence and inter-parental violence will be used when referring to studies that specifically adopt these terms.

Domestic violence and child protection is a complex, multifaceted area. It is common for domestic violence and, as specified in this case, intimate partner violence (IPV) to co-occur with other problems: ‘children’s experiences of and responses to IPV exposure cannot be viewed in isolation from other adversities and inequalities’ (Etherington and Baker, 2018, p. 70). The co-occurrence of stressful problems in early life is often referred to as adverse childhood experiences (ACEs). ACEs is a construct emerging from a long line of studies into traumatic events occurring in childhood such as domestic violence, sexual, physical and emotional abuse, household dysfunction, and neglect (Felitti et al., 1998; Dube et al., 2001). Research studies find that having these ACEs has long-lasting effects into adulthood. ACEs can be a source of long-term psychological distress as well as having longitudinal effects on physical health, substance misuse, interpersonal violence and self-harm (Hughes et al., 2017). The ‘toxic trio’ of domestic violence, substance misuse, and parental mental health problems can render children at risk of harm and complex trauma. Poverty all too frequently intersects with ACEs. Although poverty is viewed as a social marker regarding the distribution of domestic violence risk, the association is not causal (Ray, 2011). Domestic violence cuts across all socio-economic groups and all backgrounds. Victims of all backgrounds, predominantly women, face common difficulties when leaving an abusive partner. Research demonstrates that it is at the point of leaving, or after she has left, that a woman is in most danger (Calder and Regan, 2008). It is not uncommon for victims of domestic violence to remain living with perpetrators, even risking their own safety, rather than risking themselves, and their children, becoming homeless.

Teachers are well placed to play a pivotal role in identifying and responding to domestic violence since they have contact with children more than any other service. As emphasized by Sterne and Poole (2010, p. 17), ‘although staff in schools may not be able to stop the violence at home, they are in a position to make a considerable difference to children’s lives.’ Statistics from the Department for Education [DFE] (2017a) show that of the 646,120 children referred to children’s social care in England 2016–2017, the highest number of referrals, 27.5%, came from the police. The second highest percentage of referrals came from schools at 17.7% followed by health services at 14.4%. School referrals combined with education services referrals of 2.6% means that education accounted for 20.3% of referrals overall. Once referred and assessed, statistics show the percentage of children in need according to identified factors. In 2016–2017 (Department for Education [DFE], 2017a) the most common factor was domestic violence which applied to 49.9% of children in need; this incorporated violence directed at children or adults in the household. The second most common factor was mental health at 39.7% which likewise encompassed mental health of the child or adults in the household. The prevalence of domestic violence is not only high among children in need but also among the wider population with as many as one in six young people in the United Kingdom reporting experiencing it during their childhood (Radford et al., 2011).

Exposure to domestic violence generates a multitude of responses and needs and it is important for children and young people not to be regarded as a homogeneous group or lacking the capacity for posttraumatic growth and recovery: ‘it is wrong to stereotype all children as inevitably and permanently damaged by living with domestic violence’ (Mullender et al., 2002, p. 121). Although some children experiencing domestic violence will exhibit difficulties in their schoolwork, the education of others will not be adversely affected: ‘some children living with domestic abuse achieve highly in school; throwing themselves into school life and work can provide an escape’ (Sterne and Poole, 2010, p. 23). Similarly, while some students affected by domestic violence will experience educational settings as a source of continuity and security, others will experience them as challenging. It is therefore essential to take into account the range of responses to domestic violence among children. This article firstly looks at how domestic violence is conceptualized with regard to children, and how it affects them across different ages. This is followed by an examination of multi-agency working between schools and other organizations. The importance of recognizing individual and family contexts is considered before analyzing prevention education in schools. Finally, recent developments in policy and practice in the United Kingdom are examined in terms of both the challenges and opportunities they pose.

## Conceptualizing Domestic Violence in Relation to Children

Domestic violence is manifested in various ways and has been conceptualized by some as taking direct and indirect forms. Indirect abuse can result from inter-parental violence where children are not the subject of direct abuse. However, children witnessing inter-parental violence, and hearing it without necessarily seeing it, can still feel its effects: ‘While often characterized as *witnesses* to inter-parental violence, which implies a passive role, children actively interpret, attempt to predict and assess their roles in causing the violence’ (Baker and Cunningham, 2009, p. 199, emphasis in original). Indeed, the terms direct and indirect abuse have been interpreted as potentially misleading and perhaps simplistic. Callaghan et al. (2018, p. 1566) argue it is too restrictive to view domestic violence as abuse between partners in an intimate dyad whereby children are perceived as ‘affected by’ the abuse: ‘Far from passive witnesses, they are not “exposed” to violence and abuse; rather they live with it and experience it directly, just as adults do.’ Regarding children as ‘affected by’ domestic violence diminishes its impact on them. Instead, Callaghan et al. (2018) call for children to be recognized as direct victims of violence and abuse which in turn could improve professional responses to their needs.

## Impact of Domestic Violence on Young Children, Children, and Young People

Domestic violence occurs at all ages. Sterne and Poole (2010) point out that the duration of children’s encounters with domestic abuse has a greater bearing on their stress levels than the severity of the abuse. Harm caused by domestic violence can be physical, emotional, behavioral, cognitive, and social, and effects are usually overlapping and interrelated. Although harm can be present across all age phases, I will differentiate by three age groups, namely young children aged 1–4, children aged 5–10, and young people aged 11–16 since challenges and issues arising from domestic violence are different across these ages. It should be noted, however, these age groups are approximate and children’s experiences and responses will be influenced by individual needs and context.

## Impact on Young Children

The effects of domestic violence can be felt in early childhood. Research shows that psychosocial development is more problematical among toddlers exposed to IPV who additionally experience physical abuse (Harper et al., 2018). In some cases domestic violence during early childhood leads to emotional problems. Among pre-school children it can cause separation anxiety from the non-abusing parent, commonly their mother. Pre-school children’s restricted ability for coping due to their young age means that behavioral and psychological disengagement is one way they react to inter-parental violence (Baker and Cunningham, 2009). Pre-schoolers sensitized to the noise of family violence may cope by tuning out noise, consequently posing difficulties for those wishing to interact with them in the school setting. According to Baker and Cunningham (2009), pre-school children will react to inter-parental conflict in a variety of ways including becoming withdrawn, anxious, engaging in repetitive play, regressive behavior, having inhibited independence, sleep problems, tantrums or impaired understanding. The signs and symptoms of domestic violence and inter-parental violence are not always easily detectable. Moreover, it is difficult for staff in pre-school to know whether children’s conduct is associated with experience of domestic violence or regular behavior expected of this age group. If staff suspect abuse, and/or notice changes in pre-school children, background checks into the home environment will help inform their professional judgment. Staff can check if the child has a previous history of abuse and if a parent has a history of violence including toward adults or animals since they are likely to be violent toward children as well (Beckett, 2007). It is important for pre-school staff to exchange information with other healthcare professionals such as health visitors who work with children from birth to five. Guidelines in the United Kingdom recommend health visitors undertake routine screening for domestic violence and share information with pre-schools and schools as appropriate. The quality of the parent–child relationship also needs to be considered by pre-school staff, for example is the child reluctant to go home or fearful in the presence of a parent. Early years teachers and support staff can develop strategies for supporting pre-school children displaying symptoms through giving positive feedback, focusing on desirable rather than undesirable behavior, validating the child’s feelings, and preparing for transitions during the day (Baker and Cunningham, 2009).

## Impact on Children

Separation anxiety due to domestic violence is not limited to pre-schoolers and young school-aged children experiencing such anxiety could be clingy, and feign illness or be disruptive at school in the hope of being sent home. In relation to the physical impact of domestic violence Calder and Regan (2008) state effects include, but are not limited to, injury, eating problems, and stress-related conditions such as asthma and bronchitis. Emotional effects, they note, are manifested in disruption to schooling including non-attendance, attention and concentration difficulties, sleep disturbance, withdrawal, insecurity, guilt, depression and low self-esteem. Behaviorally, the impact might be changes in conduct, unpredictable behavior, aggression, anger, and hyperactivity. Being the perpetrator or victim of bullying can also ensue (Children’s Commissioner, 2018). Some children facing trauma at home display hypervigilance and hyperarousal at school, constantly watchful and fearful of danger (Sterne and Poole, 2010). Domestic violence can negatively affect cognitive skills, language development and educational attainment.

## Impact on Young People

In older children potential indicators of domestic violence include self-blame, depression, self-harm, suicidal ideation, substance abuse, risk-taking behavior, criminal behavior, poor social networks, disaffection with education, and eating disorders (Children’s Commissioner, 2018). Research indicates that experiencing domestic violence has a differential impact along gender lines. Girls are more likely to internalize symptoms in the form of withdrawal, anxiety and depression, whereas boys, though still susceptible to anxiety and depression, are more prone to externalizing symptoms through violence against peers or antisocial behavior (Baldry, 2007). Research with young people found that being listened to, taken seriously, and jointly involved in finding solutions were key means of helping them cope; in cases where no one listened, young people felt ‘doubly disadvantaged’ (Mullender et al., 2002, p. 121). The effects of domestic violence clearly have implications for student wellbeing and learning examined in more detail in the following section.

## Research With School Teachers

Research has demonstrated how domestic violence impacts on students’ engagement in learning when living within, as well as leaving, abusive homes. Those leaving domestically violent homes face the additional threat of temporary homelessness or overcrowded accommodation. Research with school teachers in England has shown that the sequential issues of domestic violence and homelessness can lead to unstable accommodation, with children being re-housed frequently, obliged to live with relatives or friends, or living long distances from school due to lack of local housing (Digby and Fu, 2017). Non-permanent accommodation has an impact in the classroom through children’s lack of ability to participate socially and academically. Primary and secondary school teachers in Digby and Fu’s (2017) sample spoke of the effects of homelessness on children they worked with such as lack of space at home to study, limited access to a computer for homework, increased anxiety and stress, and living in noisy, overcrowded accommodation which affected their sleep. The teacher participants also noted that while children in younger age groups became withdrawn, the tendency was for older pupils to exhibit anger and aggression. The study revealed the adverse effects on teachers themselves who described feeling emotionally exhausted as well as frustrated at not always being able to help their students. Children living in a refuge are additionally vulnerable to being teased and bullied at school due to the stigma associated with refuge accommodation (Sterne and Poole, 2010). Given the multiple effects of domestic violence, teachers and support staff in schools need to be equipped with knowledge, understanding and skills to identify and respond to internalized and externalized symptoms discussed next.

## Domestic Violence, Schools and Multi-Agency Working

United Kingdom government guidelines underline the importance of multi-agency working in child protection (HM Government, 2015). In order to strengthen education as part of multi-professional team working the government recently made a commitment to giving schools a greater role in forthcoming statutory guidance for safeguarding children (HM Government, 2018). Despite this emphasis ‘Surprisingly little attention has been paid to the inter-organizational information exchange in the educational context’ (Baginsky et al., 2015, p. 355) which this article seeks to examine. The United Kingdom government’s statutory guidance for schools and colleges titled ‘Keeping children safe in education’ (Department for Education [DFE], 2016) emphasizes that safeguarding children is everyone’s responsibility. Rather than being the exclusive concern of the Designated Safeguarding Lead in school ‘any staff member can make a referral to children’s social care’ (Department for Education [DFE], 2016, p. 7). However, it is evident from research and Serious Case Reviews (SCRs) into child abuse and child deaths in the United Kingdom that school staff are sometimes unclear about their role in the child protection process, and that effective training is needed to enable school staff to better support children and their parents.

Serious case reviews have repeatedly cited failure to respond to early signs of abuse, poor record keeping, and sharing information too slowly as contributing to ineffective practice (Department for Education [DFE], 2016). The SCR into the death of 4-year-old Daniel Pelka in 2012 found that recording systems in his school were not used consistently, different social work and health organizations held partial information which was not collated to enable the formation of a coherent assessment, and insufficient training of school staff resulted in their not being clear of their role in child protection, nor whom to contact with concerns (Wonnacott and Watts, 2014). Daniel was frequently hungry when he went to school where he searched for food, including in bins (Lock, 2013). Although his mother said he had health problems, he had further unexplained injuries which did not prompt a referral. His mother’s experiences of the ‘toxic trio’ of domestic violence, substance misuse, and mental ill-health complicated matters further still. Lack of professional confidence among child protection workers can be a barrier to multi-agency working as in the case of Daniel Pelka ‘where uncertainty and apprehension lead to inaction’ (Baginsky et al., 2015, p. 355). Effective child protection requires understanding of collaborative roles: ‘children are best protected when professionals are clear about what is required of them individually and how they need to work together’ (Holt, 2014, p. 56).

Collaboration across agencies is similarly examined in a report entitled ‘The multi-agency response to children living with domestic abuse’ (Office for Standards in Education, Children’s Services and Skills (Ofsted et al., 2017) which calls for health practitioners, social workers and the police to share child protection information more readily with schools. Evidence from inspections in six local authorities in England demonstrated aspects of good practice within schools for addressing domestic abuse including: schools having awareness-raising assemblies; disseminating posters and information booklets; hosting visits from charities and the police; counselors, play therapists and learning mentors working with child victims; and providing parents with support service information. The latter took the form in one school of giving out pens with a telephone number disguised as a bar code. Having support resources available in school is an important way of informing young people’s friends of how to respond to disclosure since young people experiencing violence sometimes confide in their friends (Refuge, 2008). Impediments identified by teachers in the inspection report by Ofsted et al. (2017) included limited resources for working with children affected by domestic abuse; and psychological harm being taken less seriously than physical harm. The report calls on schools to prioritize education about healthy relationships which was not always in evidence from the inspections. Schools responding to domestic violence also entails working with parents, especially mothers who tend to be the non-offending parent. Working with parents requires a context-sensitive approach which forms the focus of the following section.

## Recognizing the Child and Family Context

Parental non-disclosure of domestic violence coupled with wariness toward social services have a deep-rooted history, due in part to feelings of guilt, shame and fear of children being taken into care. Young people themselves have expressed fears of being removed from home (Ellis et al., 2015). Research shows that making a disclosure to professionals or other adults can be traumatic for children, with instances of family members becoming angry and upset and holding the child responsible for consequences (Children’s Commissioner, 2018). Cultural taboos can render disclosure of domestic violence, including ‘honor-based’ violence, even more difficult for members of certain communities. Interventions by social workers are sometimes perceived, if not directly experienced, as punitive rather than supportive. In order to facilitate identification and disclosure of domestic violence victims must be treated in a non-judgmental way and their complex needs recognized. Professionals, including teachers who are the Designated Safeguarding Lead in their school, require knowledge, training and strategies for inquiring about abuse, and how to manage both disclosures and non-disclosures.

The range of needs among those living with domestic violence requires a professional response informed by victim context. Welfare services need to adopt an intersectional approach to domestic violence and its attendant issues (Ramon, 2015) whereby disability, race and ethnicity, gender, age, socio-economic status, immigration status, and sexual orientation of children and parents alike are taken into account. Immigration status, for instance, can be a factor in non-disclosure. In recognition of the importance of intersectionality Etherington and Baker (2018) advocate service providers engage in reflexivity by examining whether their provision ignores or attends to children’s multiple social locations. The needs and access to resources of a middle-class child, for example, will differ to those of a child living in persistent or recurring poverty. An intersectional, child-centered approach is promoted by Etherington and Baker (2018), one which takes into account the specificity of children’s individual experiences, and is sensitive to the characteristics shaping their experiences. Where interconnected factors such as domestic violence and mental health problems affect a family’s context, they need to be understood and documented in conjunction with each other rather than in isolation (Lloyd et al., 2017).

## School Engagement With Domestic Violence Prevention and Education Work

In addition to making referrals to social care ‘Schools also have an essential role in educating children about domestic abuse’ (Ofsted et al., 2017, p. 28). Yet research has revealed a lack of work in school on domestic violence. A survey commissioned by the domestic violence charity Refuge (2008) involving 513 young women aged 18–21 revealed that just 13% had learned about domestic violence while at school and nearly 70% responded they would have welcomed such lessons.

Engaging in prevention education and awareness raising in school can increase domestic violence disclosure from young people, though research shows mixed outcomes, with increased disclosure following some educational programs but not others (Ellis et al., 2015). Moreover, participating in a school-based program results in some young people more likely to disclose to a family member than to professionals (Ellis et al., 2015). Trust in professionals plays a key role in domestic violence disclosure. Experiences of abuse can lead to young people having diminished trust in adults and in their ability to support and protect them, sometimes a consequence of teachers in school not acting upon student disclosure of abuse in the home (Swanston et al., 2014). Teachers building trust with young people is therefore of vital importance.

Prevention programs in school are more effective when promoted through whole-school policies and practices than through single-component programs or individual teachers (Harne and Radford, 2008). Program evaluations also show that while one-off education initiatives have some value in raising awareness of domestic violence, attitudinal change is better sustained when learning is revisited and reinforced in subsequent years (Harne and Radford, 2008). Adopting a gendered approach is another preferred format for changing attitudes as it underlines that domestic violence is rooted in unequal power relations between men and women; although men can be victims too, the majority are women and they are subject to domestic violence in more severe and repeated forms (Women’s Aid, 2009). Furthermore, where prevention programs in schools include a male facilitator, there is a higher likelihood of boys changing their attitudes (Ellis et al., 2006).

A more recent evaluation of a United Kingdom school-based domestic violence prevention program was undertaken by Fox et al. (2016). They evaluated a 6-week education program (1 h each week) delivered by domestic abuse practitioners in seven secondary (high) schools and compared participant questionnaire responses with those of participants in six schools not receiving the intervention program. The study had a total of 1,203 participants. When pre- and post-test responses were statistically analyzed, findings showed that boys and girls alike who had participated in the intervention program became less accepting of domestic violence and were more likely to seek help for abuse in comparison to those in the control group. Comparable degrees of attitude change occurred across those who had experienced abuse and those who had not experienced it. Although those in the intervention group indicated a higher likelihood of engaging in help-seeking behavior from pre- to post-test, this trend was not maintained at the 3-month follow-up data collection stage leading Fox et al. (2016) to argue that young people require more than a one-off program to persuade them of the benefits of seeking help for abuse. Congruent with previous evaluation research, Fox et al. (2016) emphasize that in order to help ensure the sustainability and effectiveness of prevention education teachers need to be trained and supported to integrate such education into the school curriculum.

For prevention and support work in school to be effective, teachers themselves evidently need to feel supported by school processes and management (Sterne and Poole, 2010). When addressing the needs of children living with domestic violence school staff should be prepared with information about services, signposting to external agencies, ensuring student safety, and knowing what to do next following disclosure. Without this information, students could be put in a worse situation than before (Howarth et al., 2016). Just as teachers need to have a clear understanding of their role in safeguarding children, so too they need to know the boundaries of their role. Research warns of the dangers of teachers acting beyond their professional scope such as asking a child to talk about their experiences without being suitably qualified which can have a traumatic effect on the child (Swanston et al., 2014). A sensitive approach is needed to help both students and their parents already living with domestic violence. Yet teachers’ responses to research reveal they often lack the professional confidence and expertise to provide domestic violence prevention education and intervention support, highlighting the need for effective staff training at both initial and continuing professional teacher education, and to include school nurses (Refuge, 2008).

The content, manner and personnel delivering domestic violence education in schools clearly require careful consideration to enhance student engagement and handle student vulnerability (Fox et al., 2014). Different models of educational program delivery have been employed in school. Some entail teachers delivering school-based initiatives themselves, others favor delivery from external specialists, while some opt for collaborative implementation. Although external facilitators have specialist knowledge, expertise and experience of discussing sensitive topics with young people, teachers have more in-depth knowledge of students and their individual circumstances (Fox et al., 2014). Working in partnership with external facilitators provides a way for teachers to develop their professional learning and confidence. The respective strengths of external specialists and teachers can be complemented through collaboration:

…preventive interventions when co-delivered with specialist organizations might offer the possibility for school staff to increase their skills in dealing with disclosures and subsequently help improve the health and well-being of children and young people. (Ellis et al., 2015, p. 60)

The need for effective professional learning and training of school staff applies to the issue of interpersonal violence too. Cross-national European research in secondary (high) schools found that teachers frequently had limited confidence and knowledge to address the problem of interpersonal violence and abuse (Barter et al., 2015). Findings from the study echoed domestic violence program evaluations in that rather than interpersonal violence and abuse being left to the efforts of an individual teacher championing the cause, the issue should be addressed at institutional level as a whole-school concern. Schools have an essential role to play, then, in tackling domestic violence and the following section examines how recent policy and practice have the potential to influence work in this area.

## Developments in Policy and Practice

Following long-running campaign calls for the introduction of mandatory relationships and sex education (RSE) in schools, the United Kingdom government announced in March 2017 it will introduce compulsory lessons in all schools in England (in Wales, Scotland and Northern Ireland RSE is expected but not compulsory). Current guidance on sex education in England was introduced in 2000 but content has not kept pace with social change, especially in respect to social media, online pornography and ‘sexting.’ While parents will still have the right to withdraw their child from sex education, draft government proposals have suggested parental withdrawal should only be possible until three terms before the child is aged 16, after which the child should be able to decide to attend (Department for Education [DFE], 2018). Subsequent to a consultation period schools will be required to teach the new RSE content from September 2020 (Department of Health, and Social Care and Department for Education, 2018). Statutory curriculum content in schools promoting healthy relationships, and raising awareness of unhealthy relationships and the unacceptability of violence in relationships, is a positive step toward equipping young people for modern-day life.

Another move in the right direction is the United Kingdom government’s consultation document which proposes the implementation of a designated senior lead for mental health in every school and college (Department of Health, and Department for Education [DOH and DFE], 2017). Educational settings present a valuable opportunity to promote mental wellbeing and prevent mental ill-health since half of all mental health conditions start by the age of 14 (World Health Organization, 2013). Although 61% of schools currently offer counseling (Department of Health, and Department for Education [DOH and DFE], 2017), concerns have been raised about schools’ ability to resource such support coupled with the length of time students sometimes need to wait. There have also been long-standing concerns around high referral thresholds for external support services such as Child and Adolescent Mental Health Services (CAMHS) which some children affected by domestic violence have not been accepted for due to referral criteria (Swanston et al., 2014). Long waiting times to access such services have been a further source of distress; the average waiting time is 12 weeks but the longest is up to 100 weeks (Department of Health, and Department for Education [DOH and DFE], 2017) during which time problems can escalate requiring more intensive and more costly support. The government’s consultation document proposes a 4-week waiting time for National Health Service mental health services for children and young people, and recommends content on mental wellbeing be part of the Personal, Social, Health and Economic education syllabus in schools. The government’s aim to implement training for the designated senior lead for mental health to all areas by 2025 has been criticized for being too slow. Concerns have been raised about the added pressure these proposals would place on an already overstretched teaching workforce facing recruitment and retention difficulties, and about the level of funding needed to ensure teachers have well-developed training for the vital role of designated senior mental health lead (Education and Health and Social Care Committees, 2018). While training programs can improve teachers’ confidence and skills to deal with children’s emotional needs (Place2Be, 2015), budgetary and workload pressures mean training opportunities are unlikely to be available in all schools, discussed further below. With the number of young people with a diagnosable mental health condition standing at one in ten (Department of Health, and Department for Education [DOH and DFE], 2017), strategies aimed at addressing the causes and symptoms of mental health problems must be adequately resourced if they are to be effective.

Another area for consideration when tackling domestic violence in children’s lives is the role of school nursing. The number of school nurses has been reduced in recent years. They are not always represented at child in need meetings, nor is relevant information always shared with them (Ofsted et al., 2017). Furthermore, term time working arrangements for school nurses mean they are not available during school holidays. Their restricted availability was referred to in the SCR of Daniel Pelka. School nurses attending relevant meetings and being employed during school holidays could facilitate greater consistency of care, better informed assessments, and improved multi-agency working.

Coming to school hungry is not conducive to learning and some schools provide breakfast and breakfast clubs. Since those living in poverty are at increased risk of domestic violence, having breakfast at school at no cost, or reduced cost, can be a valuable means to aid learning. For those impacted by domestic violence breakfast clubs can be an opportunity for quality time for parents and young children attending together (Sterne and Poole, 2010). An evaluation of breakfast clubs set up in high deprivation areas in the United Kingdom found reduced hunger in students, enhanced concentration and behavior, and improved social skills (Graham et al., 2017). Many schools offer homework clubs too. Those in temporary accommodation as a result of domestic violence may lack space or computer access to do homework and homework clubs at school can be a facilitator of learning. Extra-curricular activities and after-school clubs can also provide positive experiences. While the cost of extra-curricular activities is sometimes prohibitive, schools can provide confidential financial assistance, although some parents may be reluctant to seek financial help. Domestic abuse based on coercive control is another possible impediment to participation. Because coercive control can result in the abused parent, predominantly mothers, and their children becoming isolated and lacking opportunities for relationships with those beyond their immediate family, after-school clubs might be denied to these children but where participation is permitted they can be a means for children to develop social skills and confidence (Katz, 2016).

Paramount to effectively supporting students is the adoption of a holistic, child-centered approach. If teachers are aware of issues in students’ home lives, they will be better informed to provide tailored support to meet the individual needs of students regarding their learning, and social and emotional development. School staff need to be able to confidently ask students if anything is wrong at home and take appropriate action (Mullender et al., 2002). Research with young people affected by domestic violence found they valued teachers, tutors, learning mentors and school counselors in helping to identify abuse and access support (Howarth et al., 2016). In terms of educational attainment, additional learning support, perhaps in a one-to-one or small group context, could help improve the educational outcomes of students. This would require a sensitive approach, however, particularly as students get older and may not wish to be singled out from peers.

Early intervention strategies to help children and young people experiencing domestic violence can be strengthened through organizations engaging in joined-up thinking and working. An illustrative example is Operation Encompass, an early intervention initiative being piloted in selected areas of the United Kingdom which entails police notifying a school by 9 am if a child has witnessed or experienced a domestic abuse incident the previous evening^1^. A key adult at school (the Designated Safeguarding Lead or Deputy) is informed of the case and cascades information to teaching staff to allow immediate and ongoing support to be given to the child. As a trauma-informed charity Operation Encompass takes into account the child’s past trauma, where applicable, and the child’s responses and coping strategies. Operation Encompass can explain to the school why a child is absent or has been dropped off at school by someone else. The initiative is enabling police and schools to work in partnership to mitigate the impact of abuse and has the potential to be an exemplar of collaboration.

The Freedom Programme^2^ is another initiative being run, including in schools, to teach and empower victims of domestic violence to recognize signs of abuse and make positive changes in their lives. Organizations interested in the program need to make a commitment in terms of ensuring their staff are trained in the program and have time for implementing it. The Freedom Programme can also work with children due to start school and has proved effective in bringing about positive change in women’s and children’s lives.

Schools play a role in providing help when dealing with the fallout of domestic violence in others ways too, such as setting up practical arrangements to minimize the risk of child abduction by the offending parent following parental separation (National Children’s Home, 1994). Volunteering at their children’s school, where appropriate, can serve as a way of non-abusive parents spending more time with their children and helping to protect them (Hamby, 2014).

Perhaps unsurprisingly, some teachers feel overwhelmed dealing with issues facing both children and their parents. Pressure on teachers to address problems among children and parents, such as increasing mental health issues, have left some feeling they are becoming like social workers. Mullender et al. (2002, p. 219) emphasize the importance of teachers listening to children vulnerable to domestic violence and offering emotional support:

This is not the same as becoming social workers, which teachers understandably fear in an already over-stretched working life and without the necessary training. Rather, it means being an effective channel for children to gain access to welfare services outside of school, by opening up an early opportunity for them to confide that something is wrong.

Despite good practices taking place in schools and with partner organizations, funding cuts in the United Kingdom have meant some support services for victims of domestic violence are no longer available (Lloyd and Ramon, 2017; Ofsted et al., 2017). Survey findings from domestic violence support services in England show that 60 per cent of respondents cited funding cuts, and the associated uncertainty, as their most significant challenge (Women’s Aid, 2018). Reduced funding has led to services being unable to offer support to all women and children referred to them, loss of welfare service staff, and lower capacity to deal with increasing referrals of women with complex needs. Children and parents, predominantly mothers, living with domestic violence have been impacted by cuts to services resulting in schools taking on a greater role in supporting them. The role played by schools in supporting vulnerable children has implications for how teachers work with other agencies and in the next section I will look at how there can be tension between increased school autonomy and agencies working together.

## The Paradox of Greater School Autonomy and Working Together

Difficulties documented in research and governmental reports concerning inter-organizational working may be exacerbated by government policy devolving greater power to individual schools. Previously, state schools were funded by government and run by the local authority. Academy schools, initiated under the Labor government, and free schools under the Coalition government and subsequent Conservative government, are still state-funded but are not overseen by the local authority; they receive funding directly from central government affording them increased budgets. With budgetary independence and increased autonomy for their own governance these schools are able to set the pay and conditions for staff rather than abiding by national teacher pay and conditions required of local authority-run schools. Academies and free schools are attended by over two-thirds of secondary school students and a quarter of primary school students (Department for Education [DFE], 2017b). Despite academy and free schools still being expected to liaise closely with local authorities on matters such as child protection and safeguarding, they have greater self-determination in shaping the relationship they have with local authorities (Baginsky et al., 2015).

Academy and free school status also has a bearing on school staff training and continuing professional development (CPD) opportunities. Although local authorities are still a provider of CPD, increasingly schools are buying-in, frequently expensive, CPD and training from private providers (National Association of Schoolmasters Union of Women Teachers [NASUWT], 2018). Schools are thus operating in a market system, especially pertinent now the majority of secondary schools are academies with budgetary autonomy. Schools can choose between ‘market-leading’ training providers who offer consultancy services. Funding for school staff training comes, in part, from Pupil Premium grants given to schools in England to support the education of the most disadvantaged students. Based on rates for 2017–2018, for each student eligible for free school meals, their school will receive a payment of £1,320 (primary) and £935 (secondary). Current school practice is for the cost of staff training to be paid for by Pupil Premium and from a school’s own budget. Their budgetary independence means academy and free schools will have greater freedom to determine the nature and extent of staff training by external private providers. There have, however, been cuts in real terms in United Kingdom funding for education since 2010 (Belfield et al., 2018) posing implications for school budgets and accordingly for staff pay and training. A survey of 1,615 teachers found budgetary and workload barriers impeded their access to training: ‘Teachers report that their school does not have enough money to fund training/CPD and that external training/CPD is often very expensive’ (National Association of Schoolmasters Union of Women Teachers [NASUWT], 2018, p. 14).

Greater school autonomy has additional implications for Local Safeguarding Children Boards (LSCBs) whose role is to coordinate local work to safeguard children. As a multi-agency body LSCBs are attended by representatives from the local authority and relevant organizations such as health services and the police. However, research by Baginsky and Holmes (2015) indicates that increasing fragmentation of educational services has seen academy schools (including free schools), and private fee-paying (non-state) schools being represented on less than half of LSCBs. While over 80 percent of boards were represented at senior level by local authority schools, the same was true of only 20 percent of boards attended by academy schools (Baginsky and Holmes, 2015).

## Domestic Violence and Student Wellbeing Within an Attainment-Driven Education System

Given the spectrum of behavioral responses to domestic violence teachers need to be attuned to changes in children, some becoming withdrawn, others disruptive. Confrontational responses can, however, be difficult to account for: ‘If underlying contributory factors are not obvious or understood, those children are likely to be labeled as problematic’ (Ofsted et al., 2017, p. 14). This can lead to school staff misinterpreting students’ behavior and disciplinary action might ensue. Indeed, data show a growing number of students excluded from school have mental health needs (Education and Health and Social Care Committees, 2018), and children impacted by domestic violence (Ofsted et al., 2017) and/or living in poverty (House of Commons Education Committee, 2018) are more likely to be excluded from school in comparison to their peers. This is worrying in the context of schools focusing on examination results and league tables. Teachers and educationalists lament the marketization of education whereby examination results have become a key measure by which schools define themselves and are defined by others, and schools are set in competition with each other in the form of league tables (Berry, 2016; Berry, 2017; Scott and Scott, 2018). Research with teachers shows such changes are negatively impacting teacher-student relationships and student wellbeing, with teachers reporting having less time to attend to the needs of individual students, and reporting that their own stress levels sometimes adversely affect their interaction with students (Hutchings, 2015). Baginsky et al. (2015, p. 358) discern tension between the prioritization of examination results and children’s wellbeing:

Potentially there may be an inherent conflict between, on the one hand, pressure on institutions to demonstrate high levels of academic attainment and discipline by pupils in a competitive educational “market” and, on the other, the role of schools in recognizing and meeting the pastoral needs of children who are vulnerable or disadvantaged.

Where teacher performativity and student outcome measures in the form of examination results are at variance with the more holistic nurturing of students, efforts to support those impacted by domestic violence could be hampered and diminished. Concerns about schools becoming ‘exam factories’ have led to calls for a rebalancing of the education system whereby schools do not give precedence to academic outcomes at the expense of student wellbeing and personal development.

## Conclusion

What happens in childhood and adolescence has profound implications for wellbeing in adult life. The prevalence of domestic violence as the most common factor cited in cases of children in need in England in 2016–2017 (Department for Education [DFE], 2017a) emphasizes the need for addressing this enduring problem through prevention, early intervention and education. So too is wider attitudinal and social change needed whereby domestic violence is no longer trivialized as ‘just another domestic’ or portrayed as the fault of, predominantly women, victims, as evidenced in our earlier research into media representations (Lloyd and Ramon, 2017). Domestic violence must be addressed as a public health concern and not only as a privatized, individualized problem. The ways in which gender violence is based on and reinforced through women’s wider structural inequality and lack of power in relation to men needs to be recognized if violence within the domestic sphere is to be tackled effectively.

Encouragingly there is some evidence of domestic violence research in the context of education, though it remains relatively under-investigated. The continuing fragmentation of the United Kingdom school system and plurality of school types highlight the need for increased research to evaluate schools’ engagement in multi-agency working and to gain insight into effective practice. Some teachers and school support staff are themselves victims of domestically violent relationships and workplace support would be beneficial both for individuals and the school setting as a whole. Future research could usefully ask teachers and support staff their views on their professional learning and training needs in this important area of work.

Too frequently blame, shame and guilt cast a shadow over lives affected by domestic violence. Multi-agency working and in-school education and support can help prevent abuse and optimize outcomes for children, young people and their families living with the consequences of domestic violence.

## Author Contributions

The author confirms being the sole contributor of this work and has approved it for publication.

## Conflict of Interest Statement

The author declares that the research was conducted in the absence of any commercial or financial relationships that could be construed as a potential conflict of interest.

## Footnotes

1 http://www.operationencompass.org/

2 http://www.freedomprogramme.co.uk/
